# Consistency between chromosomal status analysis of biopsied human blastocyst trophectoderm cells and whole blastocyst cells

**DOI:** 10.1002/rmb2.12400

**Published:** 2021-06-23

**Authors:** Harunori Takahashi, Kazumasa Takahashi, Mayumi Goto, Takeo Hirakawa, Hisataka Hasegawa, Akihiro Shitara, Takuya Iwasawa, Kazue Togashi, Kenichi Makino, Hiromitsu Shirasawa, Hiroshi Miura, Wataru Sato, Yukiyo Kumazawa, Yukihiro Terada

**Affiliations:** ^1^ Department of Obstetrics and Gynecology Akita University Graduate School of Medicine Akita Japan

**Keywords:** mosaicism, next‐generation sequencing, preimplantation genetic testing, trophectoderm, whole embryo

## Abstract

**Purpose:**

This study investigated the consistency between results of preimplantation genetic testing for aneuploidy performed on trophectoderm (TE) cells and remaining blastocyst cells.

**Methods:**

TE biopsy was performed on 29 surplus cryopreserved human blastocysts. Biopsy samples and remaining blastocysts were processed using the VeriSeq PGS kit, and chromosomal statuses were compared by next‐generation sequencing.

**Results:**

Discordance was observed in the chromosomal status of 11 out of 29 blastocysts between the biopsied TE and remaining blastocysts. Concordance was observed in 11 of 12 blastocysts classified as euploid by TE biopsy and in 7 of 17 blastocysts classified as aneuploid. There was 100% concordance (7/7) in cases diagnosed as aneuploid with no mosaicism by TE biopsy. However, discordance was observed in all 10 cases showing mosaicism or partial chromosomal abnormality.

**Conclusion:**

Chromosomal status analysis based on TE biopsy does not accurately reflect the chromosomal status of the whole blastocyst. The chromosomal status is usually the same between the TE and remaining blastocyst cells in cases diagnosed as euploid or aneuploid with no mosaicism. However, mosaic blastocysts and those with other types of structural rearrangements have a higher risk of inconsistency, warranting caution during embryo selection.

## 1 INTRODUCTION

In assisted reproductive technology (ART), preimplantation genetic testing for aneuploidy (PGT‐A) of blastocysts is widely used to improve the rate of implantation and lower the rate of miscarriage by selecting euploid embryos for in vitro fertilization and embryo transfer therapy.^1^, ^2^, ^3^, ^4^, ^5^

Guzman et al reported that no more than 5 trophectoderm (TE) cells should be sampled for PGT‐A,^6^ as sampling numerous cells might damage blastocysts and increase the risk of implantation failure and miscarriage.^6^, ^7^ They also found that approximately 5 biopsied TE cells are sufficient for performing chromosomal status analysis.

Human blastocysts are constituted by approximately 300 cells;^8^ however, to this day, no study has determined the extent to which the 5 biopsied TE cells represent the chromosomal status of the whole blastocyst. As is known, TE cells differentiate into placental tissues after implantation. Therefore, knowledge of the chromosomal status of the inner cell mass (ICM), which significantly contributes to embryonic differentiation, is important. The chromosomal status of TE and ICM has been analyzed separately and compared in several studies.^9^, ^10^, ^11^ Nonetheless, these reports were based on partial biopsy of the ICM and TE and did not consider any specific associations between the outcomes of the TE biopsy and ICM chromosomal status or whole‐embryo mosaic rates. Furthermore, none of these studies managed to elucidate a clear relationship between the chromosomal status of TE biopsy samples and the group of ICM cells that will later form the fetus.^12^ Of note, invasive procedures for determining the chromosomal status of ICM are not feasible in clinical practice. Whereas up to 300 cells constitute the blastocyst, only approximately 30 Oct4‐positive cells are known to exist.^8^ Obtaining a sufficient number of ICM cells for analysis by inserting a pipette into the blastocoele is likely to result in far greater damage to the embryo and affect its development compared with a TE biopsy.

The euploid chromosomal status of ICM‐constituent cells and that of the whole embryo, including TE, are essential for implantation and the ensuing pregnancy. However, determining the chromosomal status of the whole embryo is not possible in clinical practice. Hence, the chromosomal status of the whole embryo is evaluated indirectly based on the basic findings of the chromosomal status of biopsied TE cells. The Preimplantation Genetic Diagnosis International Society (PGDIS) has provided guidelines^13^ for the use of PGT‐A in clinical practice toward the diagnosis of mosaic blastocysts. However, these guidelines have specified the criteria for using PGT‐A as an embryo screening tool and do not guarantee the concordance between the chromosomal status of TE biopsy samples and that of the whole embryo.

Despite recent reports on PGT‐A using cell‐free DNA (cfDNA) from spent embryo culture medium,^14^, ^15^, ^16^ the consistency between the actual blastocyst chromosomal status and the origins of cfDNA has been debatable. At present, PGT‐A implemented globally in clinical practice uses TE biopsy samples.

Therefore, in this study, we used next‐generation sequencing (NGS) to analyze the relationship between the chromosomal status profiles of approximately 5 biopsied TE cells and the remaining blastocyst cells from 29 frozen and thawed human blastocysts. In particular, we compared the chromosomal data obtained from biopsied TE cells and the remaining blastocyst cells to determine the consistency and association between the 2 sets of chromosomal data.

## 2 MATERIAL AND METHODS

### 2.1 Embryo source and study participant details

Twenty‐nine blastocysts obtained from 11 women were used in this study. Blastocysts were prepared and frozen as part of an ART program in our hospital between 2005 and 2011. Among blastocysts that no longer needed cryopreservation, those of patients who provided their consent were used in this study, of which 14 were frozen within 5 d postfertilization (dpf), whereas 15 were frozen 6 dpf. Blastocysts at freezing were 3AA (n = 8), 3BB (n = 2), 4AA (n = 13), 4AB (n = 1), 4BA (n = 1), 4BB (n = 2), and 6AA (n = 2) according to the Gardner classification.^17^ The mean age of patients who provided their blastocysts for freezing was 34.7 ± 2.7 years. Patients had been infertile for 4.0 ± 2.4 years, and the average number of retrieved oocytes was 2.4 ± 1.8. In all cases, oocyte retrieval was performed using the long protocol of ovarian stimulation. All embryos used underwent conventional in vitro fertilization (cIVF).

### 2.2 Embryo freezing/thawing

Blastocysts were frozen and thawed using a Cryotop Safety Kit (VT50X; Kitazato, Tokyo, Japan) and a Cryotop Safety Kit (VT50Y; Kitazato), respectively, following the manufacturer's instructions.

Embryos were transferred to Sequential Blast culture medium (Origio, Måløv, Denmark) to be recovered for 24 hours until biopsy.

### 2.3 Trophectoderm biopsy and sample stage

Trophectoderm biopsy was performed in 14 (6 dpf) and 15 (7 dpf) blastocysts. A biopsy pipette (Biopsy Flat 30°, Origio) was used to obtain 5 to 10 TE cells by suction. The biopsy pipette was then brushed onto the tip of the holding pipette (MT‐HD30, Kitazato) to separate the cells. Separated cells were collected in human tubal fluid (HTF) medium supplemented with HEPES (mHTF, HTFMS‐100, Kitazato). When the zona is attached to the embryo, the adhered sperm or cumulus cells can cause DNA contamination; hence, the zona was mechanically removed using a Piezo manipulator (Piezo Xpert, Eppendorf, Hamburg, Germany). The collected TE cell mass and remaining blastocyst portion were washed separately in fresh mHTF medium followed by treatment with 1% polyvinyl pyrrolidone (Fuji Wako, Osaka, Japan), transferred to 2.5 μL phosphate‐buffered saline (Cell Signaling Technology, Danvers, MA, USA) in a polymerase chain reaction (PCR) tube, and frozen at −20°C until further analysis. To prevent contamination by DNA fragments between samples, new tools were used for each sample during the procedure. Prior to performing the biopsy, the developmental stage of the blastocysts was classified according to the Gardner's 3‐level classification of unhatched, hatching, and hatched stages.

### 2.4 Next‐generation sequencing

Cell processing, lysis, DNA extraction, and whole genome amplification of samples were performed using a SurePlex DNA Amplification System (Illumina, San Diego, CA, USA). Following whole genome amplification pretreatment, samples were further treated using the VeriSeq PGS kit (Illumina) and then were subjected to next‐generation sequencing (NGS) analysis in a MiSeq system (Illumina). Fiorentino et al have described the protocol for using the VeriSeq PGS kit in detail.^18^ To ensure the reliability of our NGS analysis, we confirmed that the values of the parameters, overall noise, the number of total reads, and the number of reads after filtering were inside the spectrum of permitted values provided in the instruction manual of the VeriSeq PGS kit. To evaluate the concordance between the chromosomal status of the biopsy sample and the remaining blastocysts, we analyzed the obtained data using the Bluefuse Multi Software. All cases except those found to be euploid were considered abnormal. Among the noted abnormalities, only those in which 100% frequency of monosomy or trisomy was observed were judged to be aneuploid, whereas those showing less than 100% increase or decrease in the number of chromosomes were judged to be mosaic. The mosaic frequency rates of cell masses analyzed using the VeriSeq PGS kit and NGS were found to be well‐correlated with the analysis results.^19^ Thus, when the mosaic frequency rate was found to differ, the result of our analysis was considered as discordant.

### 2.5 Statistical analyses

All statistical analyses were performed using the R Project for statistical calculations (R ver. 4.0.3, Vienne, Austria). The Welch's *t* test was used to compare means of age, infertility, and number of oocyte retrievals. Fisher's exact test was used to compare the chromosomal status concordance between whole blastocysts with normal and abnormal karyotypes according to the TE biopsy. The same test was also used to compare the concordance between the chromosomal status of whole blastocysts with total aneuploidy and those with mosaic or other chromosomal status among groups with an abnormal chromosomal status. For three‐group comparisons, the data were analyzed using the Benjamini and Hochberg method. A *P*‐value of <.05 was considered statistically significant.

## 3 RESULTS

We analyzed the biopsied TE samples and remaining blastocysts using NGS and then compared their chromosomal status. The results for all samples are presented in Table 1. We found that among the 29 blastocysts, the chromosomal status of 18 (62.1%) samples was in concordance, whereas that of 11 (37.9%) blastocysts was discordant, when the 2 groups were compared.

**TABLE 1:** Overview of chromosomal concordance of all blastocysts

ID	Biopsy	Concordance	remaining blastocyst profile	TE profile	Age	Grade before freezing	Grade after recovery culture (24 h)
1	6	Yes	45, XY(−21)	45, XY (−21)	34	4AA	5BA
2	6	No	46, XY (−5 mosaic 10%)	46, XY (+5q 60%)	34	4AA	6AA
3	7	Yes	46, XY	46, XY	35	4AA	4BA
4	7	Yes	46, XY	46, XY	35	4AA	5BB
5	7	Yes	45, XX (−14)	45, XX (−14)	38	4AA	5BA
6	7	Yes	45, XY (−16)	45, XY (−16)	38	4AA	5BB
7	6	Yes	46, XX	46, XX	34	4AA	5AA
8	6	No	47, XX (+16)	46, XX (+16 mosaic 80%)	34	4AA	5AB
9	6	Yes	46, XY	46, XY	34	3AA	6AA
10	6	No	Complex chromosome abnormality	Complex chromosome abnormality	34	3AA	4BA
11	6	No	46, XX (−19 mosaic 60%)	46, XX (−4 mosaic 10%) (−19 mosaic 80%)	38	3AA	6AA
12	6	Yes	46, XX	46, XX	38	3AA	4BB
13	6	Yes	45, XY (−21)	45,XY (−21)	38	3AA	Contraction
14	6	Yes	47, XY (+4)	47, XY (+4)	38	3BB	4BA
15	6	Yes	46, XY	46, XY	35	3BB	4BA
16	6	No	46, XX	46, XX (+7 mosaic 20%) (+8 mosaic 20%) (+11 mosaic 60%) (+12 mosaic 60%) (−9 mosaic 40%)	35	4AB	Contraction
17	6	No	46, XY (3q dup 60%) (3p del 40%)	46, XY(3q dup) (3p del)	33	3AA	4BB
18	6	Yes	46, XX	46, XX	33	3AA	4BA
19	7	No	46, XX (+7mosaic 60%)	46, XX	35	4BA	Contraction
20	7	Yes	46, XX	46, XX	35	3AA	6AB
21	7	Yes	45, XY (−12)	45, XY (−12)	35	4AA	Contraction
22	7	Yes	48, XY (+9)(+17)	48, XY (+9)(+17)	34	4AA	5BA
23	7	Yes	46, XX	46, XX	34	6AA	6AA
24	7	No	46, XY (−21mosaic 80%)	46, XY(+4) (5 mosaic 20%) (+16 mosaic 80%) (−3 mosaic 20%) (−9 mosaic 20%) (−12 mosaic 20%) (−13 mosaic 20%) (−15 mosaic 20%) (−21 mosaic 80%)	33	4AA	4BA
25	7	No	46, XX (−21 mosaic 80%)	45, XX (−4 mosaic 20%) (−21)	33	4AA	5AB
26	7	No	47, XY (+21)	46, XY (+21 mosaic 80%)	37	4AA	5AA
27	7	Yes	46, XY	46, XY	37	6AA	6AA
28	7	No	46, XX (+14 mosaic 40%)	46, XX (+14 mosaic 60%)	27	4BB	non‐expand
29	7	Yes	46, XX	46, XX	27	4BB	non‐expand

Abbreviation: TE, trophectoderm.

In this study, we classified the types of concordance or discordance between the chromosomal status of biopsied TE cells and remaining blastocyst cells into 6 types. For concordance: (1) both TE and remaining blastocysts were euploid (n = 11); (2) both TE and remaining blastocysts were aneuploid (n = 7). For discordance: (3) TE was euploid, and remaining blastocyst was mosaic (n = 1, Sample ID 19); (4) TE was mosaic, and remaining blastocyst was euploid (n = 1, Sample ID 16); (5) Difference in the degree of mosaicism of only the same chromosome between TE and the remaining blastocysts (n = 3); (6) TE and remaining blastocysts presented different chromosomal abnormalities (n = 6) (Table 2).

**TABLE 2:** Summary of chromosomal status comparison between TE and the remaining blastocysts

Consistency	Number (%)	Profile	n
Concordance	18 (62.1)	Both TE and remaining blastocyst are euploid	11
		Aneuploidy in both TE biopsy samples and the remaining blastocysts	7
Discordance	11 (37.9)	Euploid TE, mosaic remaining blastocyst	1
		Mosaic TE, euploid remaining blastocyst	1
		Only the degree of mosaic of the same chromosome differs between the TE biopsy samples and the remaining blastocysts	3
		TE biopsy samples and the remaining blastocysts presented different chromosomal abnormalities	6

Abbreviation: TE, trophectoderm.

Interestingly, we only observe 1 discordant case among the 12 cases of biopsied TE cells considered normal (8.3%) and among the 10 of 17 cases of biopsied TE cells considered abnormal (58.8%), indicating a significant difference in the concordance rates obtained from the results of chromosomal status analysis between TE cells and the whole embryo (Table 3). Moreover, we observed that among the embryos for which a chromosomal status abnormality was identified in biopsied TE cells, all cases diagnosed as aneuploid with no mosaicism were in concordance with the chromosomal status of the whole embryo. However, we observed discordance between the whole‐embryo chromosomal status and the TE biopsy results in all cases with detected abnormalities such as mosaicism, a combination of mosaic, and any other chromosomal aneuploidy, and complex chromosomal abnormalities such as a partial deletion or duplication (Table 4).

**TABLE 3:** Normality of chromosomal status according to TE biopsy results and comparison with the chromosomal status of the whole embryo

Chromosomal status	Concordance	Discordance
Normal	91.7% (11/12)	8.3% (1/12)
Abnormal	41.2% (7/17)	58.8% (10/17)

**TABLE 4:** Abnormal chromosomal status detected in biopsied TE cells and a comparison of concordance with the chromosomal status of the whole embryo

Chromosomal status	Concordance	Discordance
Aneuploidy	100% (7/7)	0% (0/7)
Other Abnormality	0% (0/10)	100% (10/10)

In terms of concordance according to the developmental stage of the blastocysts during biopsy, we found that the rates for unhatched, hatching, and hatched stages were 46.2% (4/13), 71.4% (5/7), and 77.8% (6/9), respectively. Although the differences were not significant, we noticed that the concordance rates were marginally matched as the developmental stage progressed (Table 5).

**TABLE 5:** Developmental stage of blastocysts during biopsy and consistency of the chromosomal status

Developmental stage of the blastocyst	Concordance	Discordance
Un‐Hatch	46.2% (6/13)	56.8% (7/13)
Hatching	71.4% (5/7)	28.8%(2/7)
Hatched	77.8% (7/9)	22.2% (2/9)

Regarding the patient age during oocyte retrieval, we observed that the mean age was 35.1 ± 2.7 years in the concordance group and 33.9 ± 2.8 years in the discordance group; no significant difference in age was observed between the 2 groups (*P* =.2685, Welch's *t* test). In addition, we did not observe any significant difference between embryos biopsied at 6 dpf and 7 dpf, of which the concordance rates were 57.1% (8/14) and 60.0% (9/15), respectively (*P* = 1.0000, Fisher's exact test).

## 4 DISCUSSION

In this study, we used NGS to compare and analyze the chromosomal status of biopsied TE cells and remaining blastocysts. Our analysis revealed a difference in the chromosomal status between the biopsied TE cells and the whole embryo in 11 of 29 embryos (37.9%), which allowed us to confirm that the chromosomal status of TE cells does not always accurately reflect the chromosomal status of the whole blastocyst. Among the cases with discrepancies, in one case the whole blastocyst was shown to be mosaic although the biopsied TE sample was identified as euploid. Conversely, in another case, the whole blastocyst was found to be euploid although the biopsied TE sample was identified as mosaic. The distribution of cells with chromosomal abnormalities is not necessarily even in the embryo. More specifically, the proportion of abnormal cells among sampled cells does not necessarily reflect the proportion of abnormal cells in the whole embryo, which could result in an under‐ or overestimation of the mosaic frequency of the whole embryo. Such inconsistencies in the mosaic frequency between the whole embryo and that detected by TE biopsy might result in the erroneous selection of embryos that should not be transferred or exclusion of embryos that should be selected.

Popovic et al^10^ compared the chromosomal status in 3 parts of the TE and 1 part of the ICM in the human blastocyst and reported a 62.1% concordance rate in all 4 parts. Victor et al^11^ thawed embryos diagnosed as having nonmosaic aneuploidy (including partial aneuploidy) by PGT‐A and performed a rebiopsy of the ICM and TE to compare their chromosomal statuses. They found that the ICM chromosomal status matched by 96.8% the status of embryos diagnosed as having nonmosaic aneuploidy by PGT‐A, whereas only matched by 42.9% the status of those diagnosed as having partial aneuploidy. Lawrenz et al^9^ reported that an embryo diagnosed as euploid or nonmosaic aneuploid by TE biopsy has a high TE‐ICM chromosomal status concordance rate of 93.2% and 87.5%, respectively, but this concordance rate dropped to 37.5% in cases of partial chromosomal abnormalities. These studies compared the chromosomal status of TE and ICM and reported findings similar to this study in which we identified a high rate of concordance between the chromosomal status of the whole embryo and the result of the TE biopsy when the embryo was euploid or nonmosaic aneuploid. Thus, the chromosomal status of the ICM and TE of the whole embryo is suggested to be important determinants of the cytogenetic capacity of the blastocyst for pregnancy. In fact, TE mosaicism has been reported to affect the establishment of pregnancy.^2^, ^20^ Furthermore, the developmental stage of blastocysts at biopsy should also be taken into consideration. In this study, we found that the concordance of the result of the TE biopsy with the chromosomal status of the whole embryo was higher in hatching or hatched embryos than in unhatched embryos. As the embryo advances in terms of developmental stages, it is possible that embryonic cells become more cytogenetically homogeneous. Hence, studies describing the association of data obtained from biopsied TE cells with the chromosomal status of the whole embryo, including mosaicism, should provide such useful information when performing PGT‐A from TE biopsies.

In blastocysts at the TE biopsy stage, the number of TE cells is notably greater than the number of ICM cells;^8^ thus, it is possible that the results of the analysis of TE would strongly reflect the chromosomal status of the whole blastocyst. However, several studies reporting good concordance in the chromosomal status between biopsied TE and ICM^9^, ^10^, ^11^ seem to suggest that the results of TE analysis reflect the chromosomal status of the whole embryo, in cases that mosaicism is not detected. Thus, a euploid or aneuploid with no mosaicism PGT‐A result of the TE biopsy in clinical practice could be considered reliable.

However, in cases of detected mosaicism, it is important to interpret these results with caution. The PGDIS guidelines^13^ specify the following for the interpretation of mosaic results in the PGT‐A: a mosaic frequency of ≤20% is indicative of a euploid embryo; a frequency of ≥80% is indicative of an aneuploid embryo; and frequencies between these rates are indicative of mosaic embryos. In this study, discordance was observed between the biopsied TE and remaining blastocyst samples in 11 cases. When embryos were classified as euploid, mosaic, or aneuploid for each chromosome according to the PGDIS criteria, 5 cases could be considered concordant. However, there have been exceptional cases in which aneuploidy or 40‐60% mosaicism was detected in the TE, but an abnormality of the same chromosome was not observed in the whole blastocyst. Thus, the possibility of overestimating chromosomal status abnormalities in embryo screening still remains. Furthermore, it is important to note that the mosaic frequency presented by the guidelines is merely a criterion for screening. As long as an abnormal chromosomal status is detected, even at low frequencies, it is necessary to consider the possibility that abnormal cells are present in the constituent cells of the embryo.

Developing a fundamental method to solve the issue of discordance in the current PGT‐A, which is performed using just a small number of cells from the embryo, is considered to be essentially impossible. Increasing the number of biopsied cells could lower the inconsistency rates but involves a higher degree of invasiveness. Moreover, no benefits would come from increasing the number of biopsied cells, as the process would inevitably bring several negative effects such as embryo death, lower implantation rates, and higher miscarriage rates.

In our study, the concordance rate was high when no mosaicism was detected by TE biopsy, suggesting that TE biopsy is a reliable method for assessing the chromosomal status of the whole embryo. In cases of mosaicism, the consistency rates when considering the mosaic frequency were lower but could be considered high when cells were classified according to the PGDIS guidelines. However, these consistency rates were merely based on the classification criteria and did not strictly represent the frequency of abnormal cells in the actual embryo. Using the current PGT‐A method, it is difficult to accurately determine the actual chromosomal status of the whole embryo. Therefore, the development of a new technology that could replace or complement the results of analysis performed using PGT‐A, such as using cfDNA present in the culture supernatant,^14^, ^15^, ^16^ is warranted.

## DISCLOSURES

*Conflict of interest*: The authors declare that they have no conflict of interest.

## ETHICAL APPROVAL

The present study was conducted with the individual consent of each patient following approval by our Institution (Permission number: 1090.2) and the Ethics Committee of the Japan Society of Obstetrics and Gynecology (Permission number: 127). All patients donated their embryos after providing informed consent following the approval of the study protocol. Additionally, the embryos provided for this study were handled according to the regulations of the Japan Society of Obstetrics and Gynecology regarding research using human sperm, ova, and fertilized ova.
